# Supplementary material for: Effects of Obesity, Blood Pressure, and Blood Metabolic Biomarkers on Grey Matter Brain Healthcare Quotient: A Large Cohort Study of a Magnetic Resonance Imaging Brain Screening System in Japan
Source: J Clin Med. 2022 May 25;11(11):2973. doi: 10.3390/jcm11112973 (PMC9181611; doi:10.3390/jcm11112973)
Supplement: Supplementary file 1 [file jcm-11-02973-s001.zip › jcm-1688610-supplementary.pdf]

MRI acquisition parameter

The acquisition parameters were as follows: repetition time, 1800 ms; echo time, 3.7 ms; inversion time, 1100 ms; flip angle, 15° ; field of view, 256mm; voxel size, 1.0 x 1.0 x 1.2 mm for 1.5-T MR system, repetition time, 6 ms; echo time, 2.7 ms; flip angle, 8° ; field of view, 240mm; voxel size, 0.9 x 0.9 x 0.9 mm for 3.0-T MR system.

Table 2 for younger and older participants than the median

Table 2 for younger group (under or equal to 64 years old)

	mean ± standard devitation (range)	partial correlation	*p*
**blood pressure**			
systolic blood pressure	123.2±15.8 (87 - 190) mmhg	-0.1	0.011
diastolic blood pressure	74.4±11.1 (47 - 128) mmhg	-0.06	0.107
heart rate	61.8±9.7 (37 - 100) bpm	-0.1	0.011
**obesity measurement**			
body mass index	23.2±3.4 (15.82 - 48.4)	-0.25	< 0.001*
waist circumference	82.5±9.7 (20.7 - 127.6) cm	-0.22	< 0.001*
body fat percentage	24.9±6.3 (10.7 - 48.5) %	-0.2	< 0.001*
**liver function**			
total protein	7.4±0.4 (6.2 - 8.9) g/dl	0.04	0.387
albumin	4.5±0.2 (3.8 - 5.5) g/dl	0	0.981
total bilirubin	0.8±0.3 (0.2 - 3.5) mg/dl	-0.04	0.422
aspartate aminotransferase (AST)	23.8±13.8 (9 - 251) U/l	-0.16	0.002
alanine aminotransferase (ALT)	25.3±18.4 (6 - 215) U/l	-0.16	0.001*
γ-Glutamyl Transpeptidase (γ-GTP)	47.2±68.9 (7 - 1173) U/l	-0.18	< 0.001*
**renal function**			
blood urea nitrogen (BUN)	13.8±3.5 (6.5 - 55.1) mg/dl	0.09	0.067
creatinine (Cr)	0.8±0.2 (0.36 - 3.24) mg/dl	0.07	0.145

uric acid	5.4±1.3 (1.8 - 10.2) mg/dl	-0.07	0.148
**lipid metabolism**			
total cholesterol	210.9±35.6 (24 - 318) mg/dl	0.01	0.881
triglyceride cholesterol	113.6±78 (28 - 840) mg/dl	-0.05	0.155
high density lipoprotein (HDL)	64.1±16.4 (31 - 152) mg/dl	-0.04	0.274
low density lipoprotein (LDL)	122.3±33.1 (15 - 236) mg/dl	0.02	0.652
**electrolyte**			
Na	140.8±6.8 (7.2 - 146) mEq/l	0.07	0.155
K	4.3±4 (3.2 - 84) mEq/l	-0.07	0.148
Cl	102.8±2.2 (96 - 111) mEq/l	-0.02	0.703
Ca	9.4±0.4 (7.6 - 11) mEq/l	-0.02	0.696
**glycometabolism**			
fasting blood glucose (Glu)	99.2±15.4 (8 - 249) mg/dl	-0.13	< 0.001*
hemoglobin A1c (HbA1c)	5.4±0.5 (3.7 - 9.5) %	-0.08	0.023
**blood cell**			
white blood cell (WBC)	55.3±14.7 (20.5 - 169.3) $x10^2$/μl	-0.05	0.278
red blood cell (RBC)	474.6±40.2 (286 - 600) $x10^4$/μl	-0.02	0.627
hemog]obin (Hb)	14.7±1.4 (8 - 19) g/dl	-0.08	0.115
hematocrit (Ht)	43.1±3.8 (28.1 - 54.4) %	-0.11	0.034
platelet (PLT)	23.7±7.1 (5.4 - 168.6) $x10^4$/μl	0.01	0.907
fibrinogen	276.3±59.6 (140 - 630) mg/dl	0.03	0.506

* Statistical signficance with Bonferoni correction for multiple comparisons

Table 2 for older group (over 64 years old)

	mean ± standard devitation (range)	partial correlation	*p*
**blood pressure**			
systolic blood pressure	129.9±17.1 (87 - 206) mmhg	-0.08	0.075

diastolic blood pressure	72.5±10.7 (45 - 123) mmhg	-0.06	0.163
heart rate	64.3±11 (40 - 117) bpm	-0.06	0.161
**obesity measurement**			
body mass index	22.6±3 (14.22 - 32.61)	-0.16	0.001*
waist circumference	83.4±27.4 (25.9 - 860) cm	-0.03	0.460
body fat percentage	24.1±6.5 (7 - 47.6) %	-0.08	0.070
**liver function**			
total protein	7.4±0.4 (6.3 - 10.1) g/dl	-0.03	0.552
albumin	4.4±0.3 (3.4 - 5) g/dl	0.01	0.854
total bilirubin	0.8±0.3 (0.3 - 2.6) mg/dl	0.03	0.502
aspartate aminotransferase (AST)	25.5±8.7 (14 - 142) U/l	-0.08	0.093
alanine aminotransferase (ALT)	21.2±11.5 (3 - 206) U/l	-0.13	0.003
γ-Glutamyl Transpeptidase (γ-GTP)	34.9±39 (8 - 681) U/l	-0.18	< 0.001*
**renal function**			
blood urea nitrogen (BUN)	16.3±4 (5.6 - 36.7) mg/dl	0.01	0.77
creatinine (Cr)	0.8±0.3 (0.37 - 8.66) mg/dl	0.00	0.998
uric acid	5.3±1.3 (0.7 - 11.1) mg/dl	-0.03	0.501
**lipid metabolism**			
total cholesterol	211.1±67.9 (105 - 2013) mg/dl	0.08	0.041
triglyceride cholesterol	106.2±60.5 (29 - 924) mg/dl	-0.07	0.046
high density lipoprotein (HDL)	64.8±16.5 (30 - 155) mg/dl	0.01	0.812
low density lipoprotein (LDL)	117.8±27.1 (40 - 232) mg/dl	0.07	0.065
**electrolyte**			
Na	141.1±1.9 (131 - 146) mEq/l	-0.03	0.583
K	4.1±0.3 (2.6 - 5.3) mEq/l	-0.01	0.846
Cl	102.5±5 (5.7 - 111) mEq/l	0.03	0.557

Ca	9.5±3.7 (8.5 - 93) mEq/l	-0.01	0.888
**glycometabolism**			
fasting blood glucose (Glu)	105.1±22.1 (0 - 334) mg/dl	-0.10	0.005
hemoglobin A1c (HbA1c)	5.7±0.7 (4.5 - 10.9) %	-0.10	0.006
**blood cell**			
white blood cell (WBC)	61.9±231.3 (18.4 - 7010) x10^2/μl	0.00	0.925
red blood cell (RBC)	446.7±43 (241 - 596) x10^4/μl	-0,04	0.373
hemog]obin (Hb)	14±1.4 (7.7 - 18.8) g/dl	-0,08	0.072
hematocrit (Ht)	41.4±3.6 (23.6 - 54.6) %	-0.09	0.038
platelet (PLT)	21.5±4.7 (7 - 42.3) x10^4/μl	0.00	0.944
fibrinogen	308.5±65.4 (156 - 571) mg/dl	0.08	0.087

* Statistical signficance with Bonferoni correction for multiple comparisons

Table 3 for younger and older participants than the median

Table 3 for younger group (under or equal to 64 years old)

	b	Standard error	Standardization coefficient	*t*	*p*
systolic blood pressure	-0.01	0.01	-0.02	-0.69	0.49
Body mass index	-0.28	0.07	-0.11	-3.97	< 0.001*
γ-Glutamyl Transpeptidase (γ-GTP)	0.00	0.00	-0.04	-1.72	0.09
uric acid	0.24	0.19	0.04	1.30	0.19
fasting blood glucose	-0.02	0.01	-0.03	-1.12	0.26
hematocrit	-0.04	0.07	-0.02	-0.60	0.55

Table 3 for older group (over 64 years old)

	b	Standard error	Standardization coefficient	*t*	*p*
systolic blood pressure	-0.01	-0.02	-0.02	-0.42	0.678
Body mass index	-0.32	0.01	-0.12	-3.22	0.001*
γ-Glutamyl Transpeptidase (γ-GTP)	-0.03	0.01	-0.11	-2.95	0.003*
uric acid	-0.43	0.25	-0.07	-1.73	0.084
fasting blood glucose	-0.02	0.01	-0.05	-1.48	0.139
hematocrit	-0.04	0.09	-0.02	-0.41	0.681
